# Fabrication of Defect-Free Cellulose Acetate Hollow Fibers by Optimization of Spinning Parameters

**DOI:** 10.3390/membranes7020027

**Published:** 2017-06-05

**Authors:** Xuezhong He

**Affiliations:** Department of Chemical Engineering, Faculty of Natural Sciences, Norwegian University of Science and Technology, NO-7491 Trondheim, Norway; xuezhong.he@ntnu.no; Tel.: +47-7359-3942; Fax: +47-7359-4080

**Keywords:** spinning, cellulose acetate, hollow fiber membrane, orthogonal experiment design, conjoint analysis

## Abstract

Spinning of cellulose acetate (CA) with the additive polyvinylpyrrolidone (PVP) in *N*-methyl-2-pyrrolidone (NMP) solvent under different conditions was investigated. The spinning parameters of air gap, bore fluid composition, flow rate of bore fluid, and quench bath temperature were optimized based on the orthogonal experiment design (OED) method and multivariate analysis. FTIR and scanning electron microscopy were used to characterize the membrane structure and morphology. Based on the conjoint analysis in Statistical Product and Service Solutions (SPSS) software, the importance of these parameters was identified as: air gap > bore fluid composition > flow rate of bore fluid > quench bath temperature. The optimal spinning condition with the bore fluid (water + NMP (85%)), air gap (25 mm), flow rate of bore fluid (40% of dope rate), and temperature of quench bath (50 °C) was identified to make high PVP content, symmetric cross-section and highly cross-linked CA hollow fibers. The results can be used to guide the spinning of defect-free CA hollow fiber membranes with desired structures and properties as carbon membrane precursors.

## 1. Introduction

Carbon membranes have been studied in the last few years as a promising candidate for energy-efficient gas separation technology due to their improved permselectivity, thermal and mechanical stability, and chemical stability compared to those that are already used [1,2,3,4]. Hollow fiber carbon membranes are usually prepared by the carbonization of hollow fiber precursors. How to prepare cheaper, defect-free hollow fiber precursors becomes a key issue in the fabrication of carbon membranes. Many studies have reported that polymer membranes such as polyimide, polyacrylonitrile (PAN), and cellulose were used as the precursor for carbon membranes [2,5,6,7]. Cellulose is the most abundant biorenewable material with many important commercial applications. However, the potential of cellulosic materials has not been fully exploited for use as the precursors for carbon membranes because cellulose cannot be dissolved in conventional solvents due to strong inter- and intra-molecular hydrogen bonding [8]. The recently developed Lyocell process uses *N*-methylmorpholine-*N*-oxide (NMMO) to dissolve cellulose directly from biomass, and was reported to have higher efficiency compared to other processes [9], but this process embodies significant engineering challenges with regard to solvent stability, safety, and recovery. Ionic liquids are green solvents that have recently been reported to dissolve the cellulose and spin cellulose fibers [8,10,11]. However, ionic liquids are very expensive, and many of them are still not commercially available.

Fortunately, the regeneration of spun cellulose acetate (CA) hollow fiber membranes provided a potential solution to make cellulosic hollow fibers. Some studies have reported the spinning of cellulose acetate fibers [12,13,14,15,16,17,18], but mainly used in reverse osmosis (RO) and ultrafiltration (UF), and only a few of them were used as precursors for carbon membranes. Defect-free precursors are crucial for the preparation of high-performance carbon membranes. Thus, in this work, we will investigate and optimize the spinning parameters to obtain defect-free CA hollow fiber membranes.

In the present work, the well-known dry-jet wet spinning technology was used to fabricate thin and defect-free CA hollow fiber membranes. This process consists of the formation of nascent membrane, followed by the interfacial phase separation within the air gap. After that, the nascent membrane was immersed in a non-solvent (water) quench bath at a certain temperature where the phase separation occurred throughout the rest of the membranes. Many parameters, such as air gap, bore fluid composition, flow rate of bore fluid, and temperature of quench bath, etc. can affect the final fiber structure and morphology. Qin [13] and Chung [19] reported that air gap length during the spinning greatly affected the performance of membranes, and an increase in air gap resulted in a significant decrease in membrane permeation. The orthogonal experimental design (OED) method is well used in the multi-factor optimization field, and can consider the effects of all investigated parameters while significantly reducing the experimental runs. This study aims to introduce the OED method to optimize the spinning parameters. The defect-free CA hollow fiber membranes were spun under the optimal spinning conditions. The cellulosic-based membranes can be regenerated from the spun CA membranes by deacetylation treatment, and further used as precursors for carbon membranes preparation.

## 2. Materials and Methods

### 2.1. Materials

CA (*M*_W_ 100,000, average acetyl content: 39.8%) was purchased from the ACROS (Pittsburgh, PA, USA). Polyvinylpyrrolidone (PVP K30, *M*_W_ 10,000) was purchased from Sigma (Darmstadt, Germany). The solvent, *N*-methyl-2-pyrrolidone (NMP > 99.5%) was purchased from Merck (Darmstadt, Germany).

### 2.2. Spinning of CA Fibers

**Figure 1:**
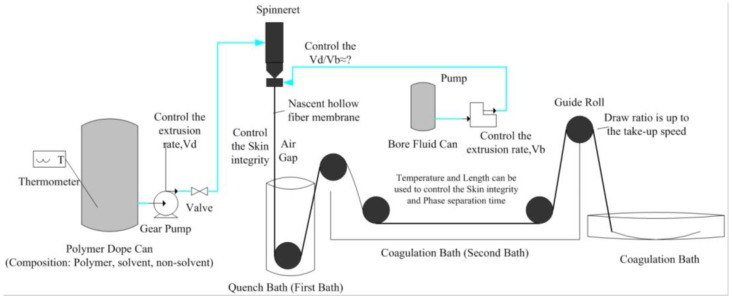
Schematic diagram of membrane spinning process.

CA hollow fiber membranes were spun using the well-known dry-jet wet spinning process [13,20]. The dope solution consisted of CA, NMP, and the additive PVP (used to increase the porosity of the carbon membrane). A schematic diagram for the spinning process is shown in Figure 1. The extrusion rate for dope and bore fluid were controlled by two gear pumps, respectively. A double spinneret (ID/OD, 0.5/0.7 mm) was used in this study with the aim of spinning defect-free hollow fibers by controlling the spinning parameters. In order to systematically investigate the effects of spinning parameters and reduce the experimental times, an orthogonal experimental design (OED) method together with multivariate analysis was introduced to optimize the spinning conditions. The factors and levels for the OED are given in Table 1, and Statistical Product and Service Solutions (SPSS) software was used to generate the experimental plan.

### 2.3. Measurement and Characterization

Fourier transform infrared spectroscopy (FTIR) spectra of the samples were obtained from the Bruker Tensor 27 FTIR instrument (Billerica, MA, USA), which was used to determine the acetyl content, PVP content, and cross-linking degree between CA and PVP in the spun hollow fibers. The morphology of spun CA hollow fibers were characterized by a scanning electron microscope (SEM) (Zeiss SUPRA 55VP, Oberkochen, Germany).

## 3. Results and Discussion

### 3.1. Experimental Results

**Figure 2:**
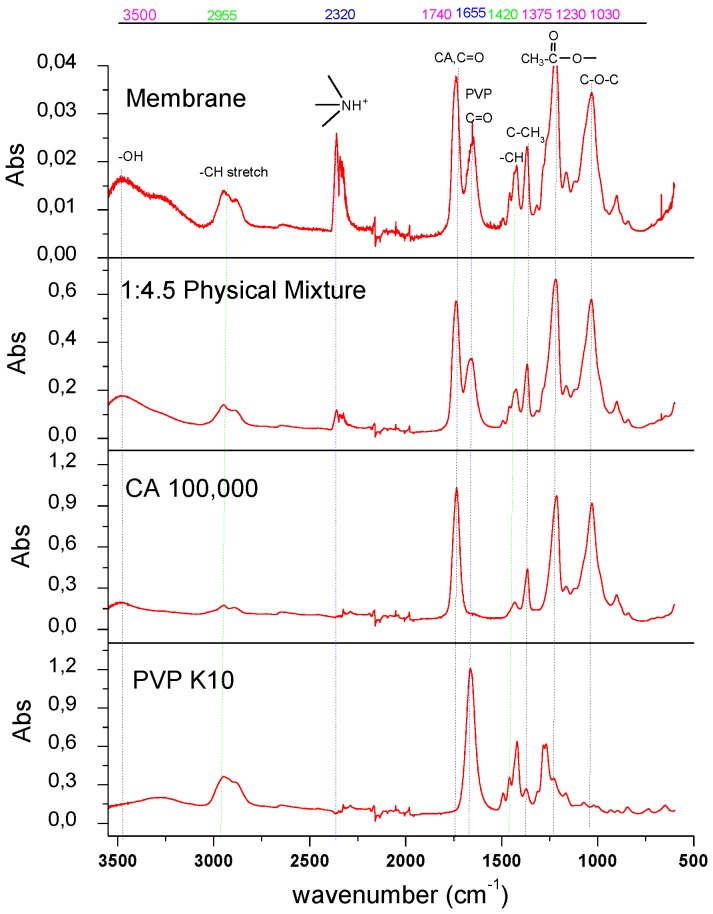
Fourier transform infrared spectroscopy (FTIR) spectra of cellulose acetate (CA) and polyvinylpyrrolidone (PVP), 1:4.5 Physical Mixture (CA and PVP power mixed) and Membrane (spun CA/PVP hollow fibers).

**Figure 3:**
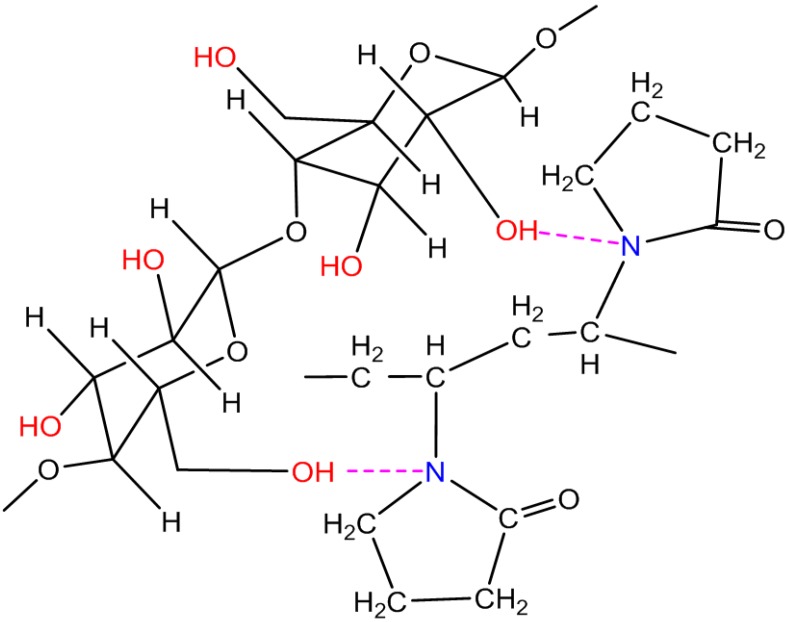
Structure for the formation of hydrogen bond between CA and PVP.

The FTIR spectra of the pure CA, the pure PVP, the physical mixture of CA and PVP, and spun hollow fiber membrane were shown in Figure 2. The characteristic adsorption peaks of 1030 cm^−1^, 1230 cm^−1^, and 1740 cm^−1^ are attributed to the ether group ($\nu_{C-O-C}$), acetyl ester group ($\nu_{{\mathrm{CH}}_{3}-C=O}$), and carbonyl group ($\nu_{C=O}$) of CA, respectively. The peak at 1665 cm^−1^ is attributed to the carbonyl group in the PVP. Moreover, the assumption that an additional hydrogen bond may form between the CA and tertiary amide group of PVP is possible because the IR spectrum displays a strong absorption band in the region of 2250–2700 cm^−1^ that is characteristic of hydrogen bond for tertiary amide [21], as illustrated in Figure 3.

**Figure 4:**
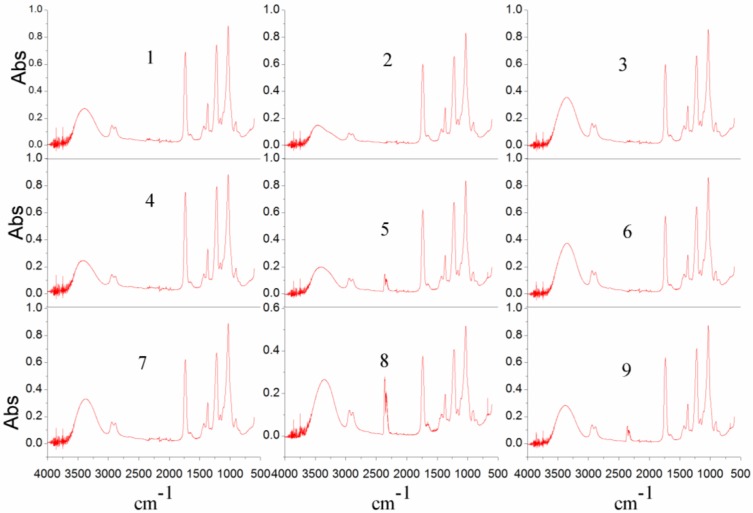
FTIR spectra of the spun hollow fiber membranes for the experiment plan, which were shown in Table 2.

**Figure 5:**
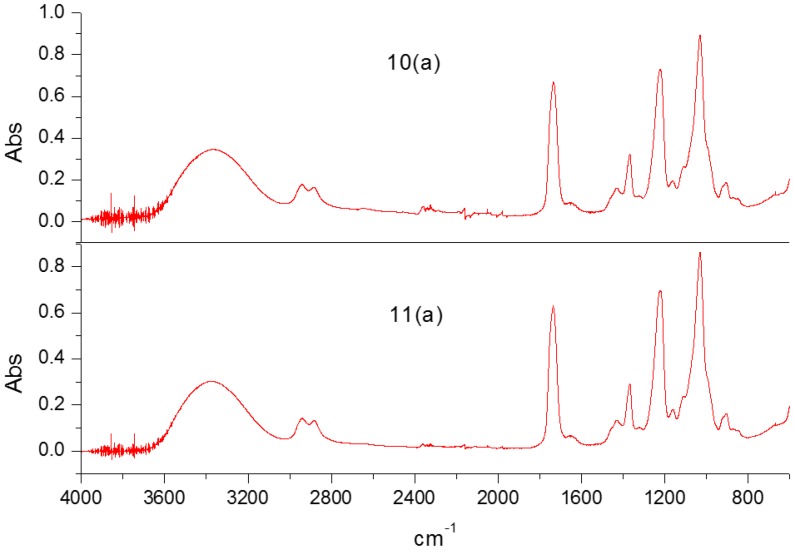
FTIR spectra of spun membranes for holdout experiments, which were shown in Table 2.

The FTIR spectra for spun membranes of OED are shown in Figure 4 and Figure 5. The absorption ratios of A2320 cm^−1^/A1030 cm^−1^ and A1665 cm^−1^/A1030 cm^−1^ were used to characterize the cross-linking degree and the PVP content in the membrane, respectively. The cross sections of membrane morphology were characterized by SEM. Table 2 gives the OED results for different spinning conditions.

### 3.2. Conjoint Analysis

The conjoint analysis in the SPSS package was used to analyze the results of the orthogonal experimental design [22]. The utilities (part-worth) reflect the importance of each factor level. The range (highest minus lowest) of the utility values for each factor provides a measurement of how important the factor was to overall preference [23]. Factors with larger utility ranges play a more significant role compared to those with smaller ranges. The importance score (IMP) of factor *i* (%) is calculated as:

(1) $$IMP_{i}=100\frac{Range_{i}}{\sum_{i=1}^{p}Range_{i}}$$

where *p* = factor number. If several subjects are used in the analysis, the importance of each factor is separately calculated for each subject, and then averaged. For the prediction, the probability of each simulation (*p_i_*) can be estimated according to the three different methods: (1) The maximum utility model determines the probability as the number of respondents predicted to choose the case divided by the total number of respondents; (2) The BTL (Bradley–Terry–Luce) model determines the probability as the ratio of one case utility to that for all simulation cases; and (3) The logit model is similar to BTL, but uses the natural log of the utilities instead of the utilities. The conjoint analysis method reported in our previous work [23] was used to estimate the part worth of the contribution from each factor’s level in this work, and the response is the combination of three subjects (the PVP content, cross-linking degree, and membrane morphology; these parameters will accordingly influence the microporosity, mechanical strength, and structure and morphology of carbon membrane). The importance of each factor was calculated separately for each subject, and then averaged. The correlations of Pearson’s R and Kendall’s τ were 0.964 and 0.957, respectively, which indicated a good consistency between the estimated results from the model and the experimental data. Table 3 shows the utilities (part-worth) of each factor level, and averaged importance score of each factor. Higher utility values indicate greater preference in the selection of spinning condition.

The range of the utility values (averaged importance score) for each factor provides a measure of the importance of each factor to the overall performance. Factors with greater averaged importance score play a more significant role than those with smaller values. From Table 3, one could find that the importance of these four factors was sorted as follows:

• air gap > bore fluid composition > flow rate of bore fluid > quench bath temperature.

It can be clearly seen that air gap was the most important parameter of the spinning process, which kept a good consistency with the previous results [13,19]. Therefore, the length of air gap needs to be well controlled during the spinning process to prepare defect-free CA hollow fiber membranes with desired structure and property. Moreover, all the utilities are expressed in a common unit, and can be summed to give the total utility of any combination. Table 4 shows the comparison between an arbitrary selected combination of factor level and the optimal spinning condition. It can be found that utility of the optimal condition (Case 2) is much higher compared to Case 1.

### 3.3. Predictions

**Figure 6:**
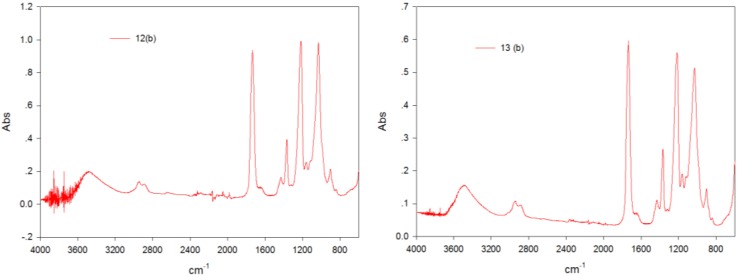
FTIR spectra for simulation cases, which has been mentioned in Table 2.

The real power of conjoint analysis is to predict the performance (structure and property) of the spun hollow fibers that have not been investigated in the experiments—the simulation cases (see No. 12 and 13 in Table 2). The simulation results are given in Table 5. It was found that the utility of Case 2 was larger than that of Case 1 across the three response variables (PVP content, cross-linking degree, and membrane morphology) in this study. All three models—maximum utility, Bradley–Terry–Luce (BTL), and logit—indicated that simulation Case 2 would be preferred. In order to validate this simulation result, CA hollow fiber membranes were spun under these two conditions and characterized by FTIR, as shown in Figure 6. The PVP content (A1665 cm^−1^/A1030 cm^−1^) was higher and cross-linking degree was weaker for Case 2, which indicated that the membrane spun from Case 2 was better for use as a precursor for carbonization. The predicted score of Case 2 was also higher compared to Case 1. Thus, the generated model from the conjoint analysis can be used to guide the spinning of hollow fibers with the desired structure and properties.

## 4. Conclusions

CA hollow fiber membranes were spun from a dope solution containing (CA + PVP)/NMP at different conditions using a dry-jet wet spinning process. The orthogonal experimental design method was firstly used for the optimization of spinning conditions. The experimental results showed that the importance of these four factors was sorted as:

air gap > bore fluid composition > flow rate of bore fluid > quench bath temperature.

The spinning parameter *air gap* was identified as the most important factor during the spinning process, which kept the consistency with the results reported in the literature. Moreover, the optimal spinning condition with a bore fluid composition (water + NMP (85%)), an air gap (25 mm), a flow rate of bore fluid (40% of dope flow rate), and a temperature of quench bath (50 °C) was obtained for making cellulose acetate hollow fibers with high PVP content, symmetrical cross-section, and high cross-linking degree. The proposed OED method can be well used for the optimization of spinning conditions, and the simulation results based on conjoint analysis can be applied to guide the spinning of hollow fibers with desired structure and properties.

## Figures and Tables

**Table 1:** Factors and levels for orthogonal experimental design (OED) of spinning conditions.

Level	Bore Fluid Composition	Air Gap	Flow Rate of Bore Fluid *	Quench Bath Temp.
1	H_2_O	15 mm	20%	20 °C
2	H_2_O + NMP (85%)	25 mm	40%	50 °C
3		35 mm	60%	

* 100% means the same flow rate as the dope flow rate.

**Table 2:** The OED results for the optimization of spinning parameters.

No.	Bore Fluid	Air Gap (mm)	Flow Rate of Bore Fluid (%) *	Quench Bath Temperature (°C)	Cross-Linking Degree ^c^	PVP Content (%)	Membrane Morphology
1	water	15	40	20	5.19	9.41	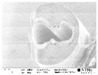
2	water	35	20	50	3.14	9.01	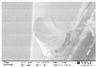
3	water + NMP (85%)	15	60	50	3.68	9.11	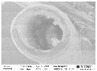
4	water	25	60	20	5.63	10.08	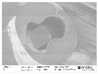
5	water + NMP (85%)	35	40	20	8.61	8.59	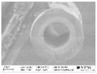
6	water + NMP (85%)	25	20	20	3.03	8.16	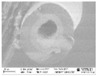
7	water	25	40	50	4.07	9.41	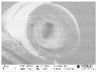
8	water	15	20	20	21.47	10.83	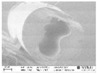
9	water	35	60	20	7.34	7.75	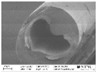
10(a)	water	15	60	20	2.79	7.72	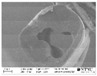
11(a)	water + NMP (85%)	25	60	20	7.17	10.37	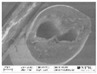
12(b)	water	25	40	20			
13(b)	water + NMP (85%)	25	40	20			

(a): Holdout; (b): Simulation; ^c^ hydrogen bond between CA and PVP, the value is calculated according to the ratio of absorption from IR spectra (A2320 cm^−1^/A1030 cm^−1^); * Percentage of the dope flow rate. NMP: N-methyl-2-pyrrolidone.

**Table 3:** Utilities and averaged importance scores for different factors.

Factors and Levels		Utility	Averaged Importance Score (%)
Bore fluid composition	water	−0.917	28.731
	water + NMP(85%)	0.917	
Air gap	15 mm	−1.111	29.467
	25 mm	0.889	
	35 mm	0.222	
Flow rate of bore fluid	20%	−0.778	27.860
	40%	0.889	
	60%	−0.111	
Quench bath Temp.	20 °C	−0.750	13.942
	50 °C	0.750	
(Constant)		5.556	

**Table 4:** An example for combination of different spinning conditions.

Case	Utility					Total Utility	Remarks
	Bore Fluid Composition	Air Gap	Flow Rate of Bore Fluid	Quench Bath Temp	Constant		
1	water (−0.917)	35 mm (0.222)	60% (−0.111)	20 °C (−0.75)	5.556	4	
2	water + NMP (85%) (0.917)	25 mm (0.889)	40% (0.889)	50 °C (0.75)	5.556	9.001	Optimal

**Table 5:** Simulation results by conjoint analysis. BTL: Bradley–Terry–Luce.

Card Number	Score	Maximum Utility(a)	BTL	Logit
1	5.667	33.3%	43.7%	26.9%
2	7.500	66.7%	56.3%	73.1%
